# Biohydrogen and Bioethanol Production from Biodiesel-Based Glycerol by *Enterobacter aerogenes* in a Continuous Stir Tank Reactor

**DOI:** 10.3390/ijms160510650

**Published:** 2015-05-11

**Authors:** Rujira Jitrwung, Viviane Yargeau

**Affiliations:** Department of Chemical Engineering, McGill University, 3610 University Street, Montreal, QC H3A-0C5, Canada; E-Mail: jitrwung@mail.mcgill.ca

**Keywords:** biohydrogen, bioethanol, crude glycerol, *Enterobacter aerogenes*, CSTR

## Abstract

Crude glycerol from the biodiesel manufacturing process is being produced in increasing quantities due to the expanding number of biodiesel plants. It has been previously shown that, in batch mode, semi-anaerobic fermentation of crude glycerol by *Enterobacter aerogenes* can produce biohydrogen and bioethanol simultaneously. The present study demonstrated the possible scaling-up of this process from small batches performed in small bottles to a 3.6-L continuous stir tank reactor (CSTR). Fresh feed rate, liquid recycling, pH, mixing speed, glycerol concentration, and waste recycling were optimized for biohydrogen and bioethanol production. Results confirmed that *E. aerogenes* uses small amounts of oxygen under semi-anaerobic conditions for growth before using oxygen from decomposable salts, mainly NH_4_NO_3_, under anaerobic condition to produce hydrogen and ethanol. The optimal conditions were determined to be 500 rpm, pH 6.4, 18.5 g/L crude glycerol (15 g/L glycerol) and 33% liquid recycling for a fresh feed rate of 0.44 mL/min. Using these optimized conditions, the process ran at a lower media cost than previous studies, was stable after 7 days without further inoculation and resulted in yields of 0.86 mol H_2_/mol glycerol and 0.75 mol ethanol/mole glycerol.

## 1. Introduction

Due to environmental concerns and an expected shortage of petroleum fuels, alternative energy sources such as bioethanol, biodiesel, and biohydrogen are being increasingly researched. Biodiesel is an alternative fuel to diesel oil, and there has been a slow increase in the number of biodiesel plants for several reasons, such as increasing price of raw materials (vegetable oil or animal fats and alcohols) [1], and the decreasing and fluctuating market price of biodiesel by-products, mainly glycerol [2]. Based on the stoichiometry of the trans-esterification reaction occurring in biodiesel production, crude glycerol (biodiesel-based glycerol) is formed as a by-product and represents approximately 10% by weight of the biodiesel produced. As the number of biodiesel plants is growing, increasing amounts of crude glycerol are being generated, which has been affecting glycerol’s market price and biodiesel production economics. To mitigate this impact, various approaches to valorize crude glycerol have been studied, including direct burning as heating oil, purification for sale as commercial glycerol, steam reforming to make hydrogen, and microbial conversion into hydrogen. The first three options consume more energy and are less cost-effective and, in the case of direct combustion, require off-gas treatment to control the emission of toxic gases. Because crude glycerol is contaminated with many chemicals from the biodiesel process, purification is not economically preferable. Auto-thermal reforming of the crude glycerol yields hydrogen but emission of greenhouse gases is a concern [3,4]. Microbial fermentation has therefore been identified as a promising alternative, considering that the use of optimal conditions and proper microorganisms can favor the metabolic pathways leading to the desired products (biohydrogen and bioalcohols) while minimizing the formation of other side-products.

Studies of biohydrogen production from glycerol were first summarized by Nandi and Sengupta [5] and then by Willke and Vorlop [6]. Studies reported the use of *Klebsiella pneumonia* [7,8,9], Clostridium species such as *C. acetobutylicum* [10], *C. butyricum* [11,12], *C. pasteurianum* [13,14], and *Escherichia coli* [15]. The main side-product produced by these strains is propanediol (PDO) which has a wide range of applications in polymer fields for production of polyester, polypropylene terephtalate (PPT), polyethylene terephtalate (PET) and polyurethane (PU) [16]. However, the grade of purification of PDO for such applications has to be from 95% to over 99%, which requires energy-consuming recovery steps [17]. In the last two decades, bioethanol has also been the subject of increasing research as a desired product of biohydrogen production. Bioethanol obtained from the fermentation of waste streams such as crude glycerol is cheaper to produce than bioethanol obtained from yeast fermentation of feedstock such as cassava, wheat, and corn [18]. In addition, bioethanol obtained as a side-product of the conversion of the glycerol-containing waste into biohydrogen could be returned to biodiesel processes and used as raw material as part of an integrated biofuel process [19]. More recently, various researchers confirmed the potential of *Enterobacter aerogenes* for the conversion of crude glycerol into biohydrogen and bioethanol [5,18,19,20,21,22,23,24,25]. They have reported hydrogen yields in the range of 0.84 to 1.12 mole/mole GL, ethanol yields ranging from 0.79 to 1.04 mole/mole GL, and glycerol conversion of 93% to 100%. However the knowledge developed at the lab batch scale has yet to be scaled-up and transferred to the continuous operation mode required for the development of industrial applications.

The two main types of bioreactor that have been being studied in order to identify the configuration and operating parameters that would enhance the desired products yields, while inhibiting the side-reactions, are the continuous stir tank reactor (CSTR) and the packed bed reactor (PBR). CSTR is widely used because of the ease of control of the operating parameters [26,27,28]. On the hand, PBR is commonly studied for its known ability to enhance cell density and, as a result, its increased rate of hydrogen production [17,29]. Although PBR is superior to CSTR in supporting a faster conversion rate, PBR requires regeneration of the catalyst or bed used. Due to the known tendency of *E. aerogenes* to self-flocculate [30] and considering the higher risk of clogging of PBRs, CSTR was considered a better option.

Using the optimized minimum mineral synthetic media composition, inoculum volume ratio and oxygen concentration reported by Jitrwung *et al.* [19,23] for the conversion of glycerol by *E. aerogenes*, the objectives of this research were: (1) to scale-up the batch process from 60 mL to 1.8 L; (2) to use the optimal conditions in continuous mode and optimize the CSTR operating parameters including feed rate, liquid recycling ratio, pH, mixing speed, glycerol concentration, and waste recycling; and (3) to test the stability of the process over time when operated at the optimized conditions.

## 2. Results and Discussion

### 2.1. Scale up of Batch Fermentation

**Figure 1:**
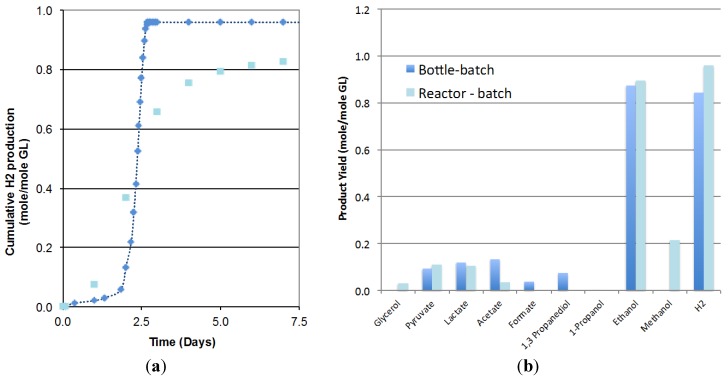
Comparison of (**a**) the hydrogen production; (**b**) the product formation, in the 3.6-L bioreactor (♦, Reactor batch) with 125-mL serum bottles (■, Bottle batch) (2 replicates).

Figure 1a compares the biohydrogen production obtained in the 3.6-L bioreactor operated in batch mode to the one obtained at the smaller scale (125-mL bottles). Results showed that due to scale-up effects, the lag phase increased from 0.8 to 2.0 days but the rate of hydrogen production increased from 0.29 to 0.80 mole/mole GL/Day and the hydrogen yield increased from 0.84 to 0.96 mole/mole GL. The analysis of the final liquid composition, presented in Figure 1b, indicated similar ethanol yield at both scales (0.90 compared at larger scale compared to 0.88 mole/mole GL) and glycerol conversions higher than 99% in both cases. The lower production of acetate, 1,3-propanediol and formate obtained in the bioreactor seems to indicate a shift in the metabolism at a larger scale that would enhance the metabolic pathways leading to the desired products, hydrogen and ethanol and the possible conversion of formate to CO_2_. No significant differences in pyruvate and lactate production were observed. As a result, initial glycerol concentration, inoculum volume and media composition, previously optimized in small bottles, were considered applicable to the scaled-up batch system.

**Figure 2:**
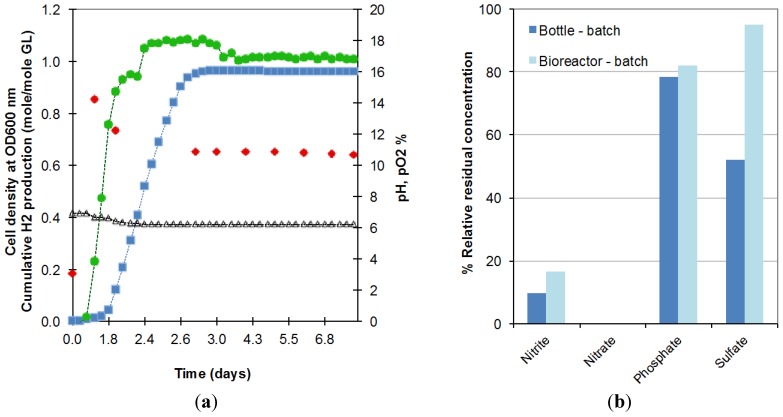
(**a**) Evolution in the batch bioreactor of cell density (♦), cumulative biohydrogen production (■), pH (∆), pO_2_ (●) and; (**b**) anion consumption, during the fermentation of 18.5 g/L crude glycerol in bottles (Bottle-batch) and bioreactor (Bioreactor-batch) (2 replicates).

The longer lag phase might be explained by the larger amount of oxygen still dissolved in the liquid contained in the bioreactor due to the difficulty of decreasing the initial oxygen concentration in this larger and more complex system. This oxygen prolonged the period of time over which *E. aerogenes* expanded its population under semi-anaerobic conditions before moving to anaerobic conditions, under which oxygen from decomposable salts is used and hydrogen production starts. As shown in Figure 2a, in the first period of 6 h, pO_2_ (circles) remained at 0% while the optical density went from 0.20 to 0.87 (at 600 nm). When dissolved oxygen was completely consumed, oxygen was released from the consumption of salts resulting in rising pO_2_, decreasing cell density and commencement of biohydrogen production. This hypothesis of higher dissolved oxygen availability in the reactor is also supported by the higher residual concentration of anions obtained in the bioreactor operated in batch mode (Figure 2b).

The faster production rate obtained in the bioreactor might be attributed to the better mixing. The mixer used was a 2-blade turbine agitator set at 500 rpm. This mixing was more efficient than the one obtained in the incubator-shaker set at 120 rpm. This maintained homogeneous conditions and maximal dissipation to the headspace of the hydrogen produced. The effect of mixing speed in the bioreactor is discussed in Section 2.2.4.

### 2.2. Optimization of the CSTR

The CSTR was operated over a period of 1 month including a 3-day start-up in batch mode in order to achieve a H2 concentration of 70%–80%, and then switching to continuous mode by feeding fresh glycerol-containing media (data not shown). When the stationary phase was reached, the fresh feed rate (FR), liquid recycling ratio (LR), pH, mixing speed, glycerol concentration, and waste recycling were varied as presented in the following sections, one parameter at a time, until a new steady state was reached in order to identify the optimal operating conditions.

#### 2.2.1. Feed Rate

**Figure 3:**
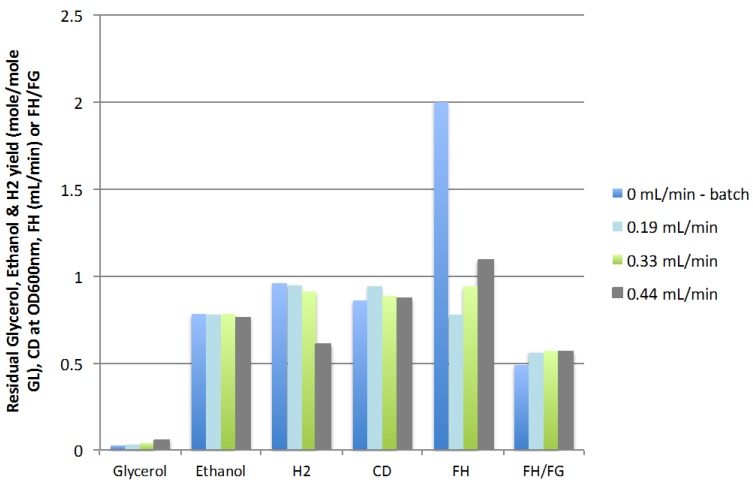
Effect of feed rate on residual glycerol, ethanol and hydrogen yields, cell density, rate of hydrogen production (FH), and mole ratio of H_2_/gas (FH/FG) (3 replicates).

The results presented in Figure 3 show that increasing the feed rate of fresh glycerol-containing media (FR) from 0 (batch) to 0.44 mL/min caused a slight increase in residual glycerol from 0.03 to 0.06 mole/mole GL and a reduction in hydrogen yield, from 0.96 to 0.61 mol/mol GL, while cell density (CD) and ethanol yield remained almost constant. The ratio of hydrogen in the gas produced (FH/FG) remained constantly above the value obtained in batch mode (0.56 *vs.* 0.49, respectively). The maximum rate of production of hydrogen (FH) obtained in the continuous mode, 1.09 mL H_2_/min obtained at a feed rate of 0.44 mL/min (corresponding to a dilution rate of 0.0148 h^−1^), was significantly lower than the rate observed in batch mode (2.00 mL H_2_/min). Although the feed rate of 0.44 mL/min resulted in a lower hydrogen yield, this feed rate of 0.44 mL/min yielded the highest rate of hydrogen production in the continuous mode of operation and it was hypothesized that the optimization of the other CSTR operating parameters would compensate for the negative impact on hydrogen yield of this fresh feed. The maximum feed rate tested, 0.44 mL/min (0.0148 h^−1^ dilution rate), was thus used for the optimization of the other parameters.

#### 2.2.2. Liquid Recycle Ratio (LR)

**Figure 4:**
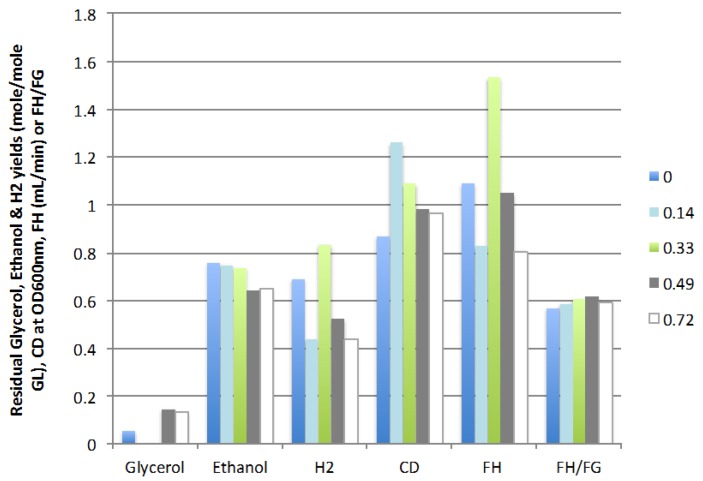
Effect of liquid recycling ratio on residual glycerol, ethanol and hydrogen yields, cell density, rate of hydrogen production (FH), and ratio of H_2_/gas (FH/FG) (3 replicates).

Figure 4 shows that the optimal ratio of liquid recycle is 0.33 at which 1.53 mL H_2_/min was produced for an hydrogen yield of 0.83 mole/mole GL and an ethanol yield of 0.74 mole/mole GL. Further increases in the recycle ratio resulted in lower cell density and glycerol conversion as well as decreased H_2_ and ethanol production. The optimal liquid recycling ratio partially compensated for the dilution effect of the feed rate (continuous *vs.* batch mode) but was not sufficient to bring the yield and rate of production of hydrogen back to the values obtained in the batch mode (0.96 mole/mole GL and 2.00 mL H_2_/min). However, at these conditions the microbial population in the CSTR was sustainable without further inoculation and continuous and stable production of hydrogen and ethanol was obtained, two significant advantages of the continuous operation mode.

#### 2.2.3. pH

The pH range tested was selected based on preliminary experiments performed in batch mode and indicating an optimal pH range of 6.2 to 6.6 (data not published) for hydrogen production, the optimal growth condition given in the ATTC guidelines for the strain ATCC 35029 (pH 6.8) and the optimal pH values reported in literature for *E. aerogenes*: Yokoi *et al.* [31]: 6.0–7.0, Fabiano and Perego [32]: 6.1–6.6 and Jo *et al.* [33]*:* 6.13. Figure S1 shows that increasing pH from 6.2 to 6.6 enhanced the cell density but the lowest pH (6.2) and highest pH (6.6) tested resulted in reduced H_2_ yield and rate of production. The formation of significant amount of 1-propanol at these conditions suggests that lower or higher pHs might favour different pathways reducing the amount of hydrogen produced.

#### 2.2.4. Mixing Speed

Mixing speed significantly influences bacterial growth, glycerol conversion and H_2_ production as shown in Figure S2. Rachman *et al.* [34] observed that increased mixing speed inhibits the self flocculating nature of *E. aerogenes*. This explains the decrease in cell density and lower glycerol conversion and hydrogen production obtained at higher mixing speeds. On the other hand, using a lower mixing speed (300 rpm) also affected the cell density and caused *E. aerogenes* to flocculate too much (visual observations). Under these conditions, significantly lower glycerol conversion was observed and the metabolism shifted towards production of 1-propanol and acetic acid, as shown in Figure S2.

#### 2.2.5. Glycerol Concentration

The optimal conditions determined in the previous sections (FR 0.44 mL·min^−1^, LR 0.33, pH 6.4, and 500 rpm) were used to test the effect of glycerol concentration on hydrogen and ethanol production. The results shown in Figure S3 indicate that an increase in glycerol concentration reduced its conversion and resulted in much higher concentrations of intermediates, such as 1,3-propanediol and 1-propanol, and lower hydrogen and ethanol yield. Higher glycerol concentration increased cell density but inhibited the H_2_ production. This might be caused by substrate inhibition or due to negative effects of other crude-glycerol constituents, as reported by Ito *et al.* [20].

#### 2.2.6. Waste Recycling

To make this process more economical, the possibility of replacing a fraction of the feed of fresh media with a recycled stream of waste was tested. The waste solution recycled contained low residual concentrations of salts and nutrients, except for Na_2_HPO_4_, which contributes the most to the cost of the media due to its higher proportion (88 wt % of added salts). It was assumed that salts such as NH_4_NO_3_, MgSO_4_, FeSO_4_, and Na_2_EDTA were sufficiently consumed by *E. aerogenes* to be replenished. The feed rate to the CSTR was thus maintained constant at 0.44 mL/min while its composition was modified by mixing the waste solution with the crude glycerol-containing fresh media (without Na_2_HPO_4_) at mass ratios of 0%, 33% and 66%. The results presented in Figure S4 (Supplemental material) shows that increasing waste recycling resulted in significant reduction in biohydrogen and bioethanol production even though glycerol conversion was not affected by waste recycling. This can be explained by a shift in metabolism towards the formation of 1,3-propanediol and 1-propanol. This metabolic shift might be due to the increased concentration of organic acids such as lactic and acetic acids.

### 2.3. Testing of Stability of Production in Continuous Mode—Process Stability

**Figure 5:**
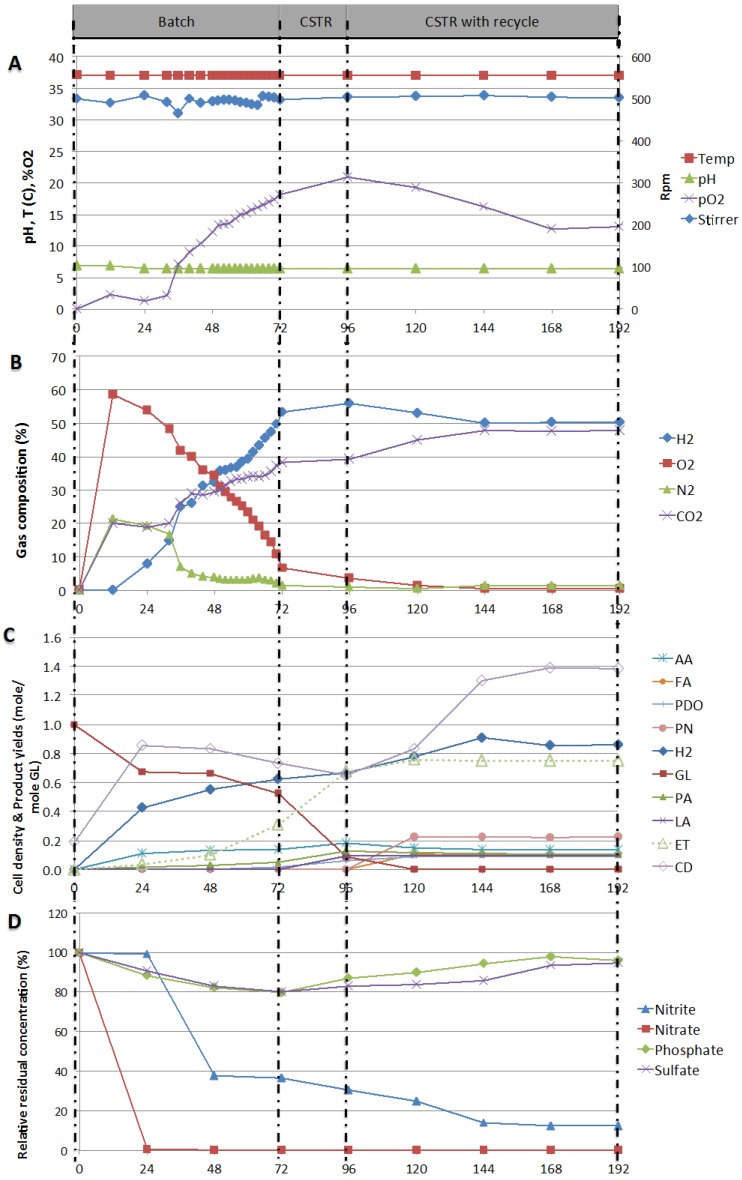
(**A**) Process parameters: pO_2_, Mixing speed (rpm), Temperature (°C), and pH; (**B**) Gas compositions: H_2_, O_2_, N_2_, and CO_2_; (**C**) Cell density (CD), residual glycerol and products yields; (**D**) Percent relative residual concentration of anions, with respect to time over the three mode of operation batch (I), continuous (II) and continuous with recycling (III).

The optimized conditions of the process, determined in the previous sections, were used to test the stability of the continuous system. The system was run in three consecutive modes, batch (I), continuous (II) and continuous with recycle (III). After 72 h in the batch mode (I), 70% to 80% glycerol conversion was reached and the feed of fresh glycerol-containing media was started in order to switch to the continuous mode. At 96 h, the CSTR operation was stable but cell density was decreasing. Liquid recycling was then started and constant product concentrations were obtained at 144 h onwards. The stable operation of the CSTR with recycling was maintained for two days (until 192 h of operation). Figure 5 presents various variables over time for the three modes of operation tested and delimited on the figure by vertical dashed lines. Figure 5A presents the controlled variables, temperature and mixing speed, as well as pH and pO_2_, Figure 5B shows the gas composition, Figure 5C the cell density and product yields, and Figure 5D presents the relative residual concentration of the anions.

During the first of 12 h of the batch operation, pO_2_ was rising (Figure 5A) as nitrate was reduced (Figure 5D). The concentration of nitrite did not vary over this period of time, probably because the rate of nitrite production was balanced with the rate of nitrite consumed. This hypothesis is supported by the gas composition (Figure 5B) over the same period of time, O_2_ and N_2_ concentrations increased to 60% and 20%, respectively, indicating that *E. aerogenes* digested nitrate and nitrite contained in solution.

**Figure 6:**
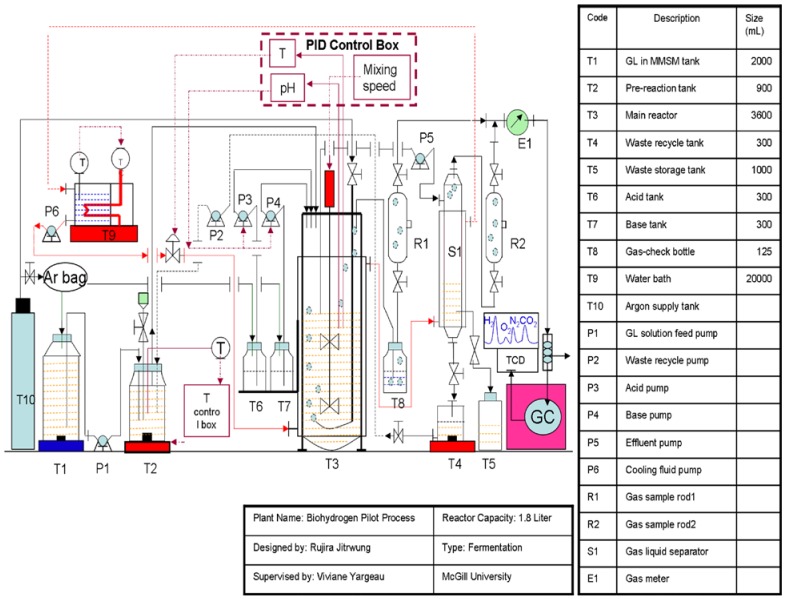
Continuous biohydrogen production set-up.

After the first 12 h, H_2_ production started and *E. aerogenes* started digesting glycerol into acids, as suggested by the metabolic pathways reported by Gonzalez *et al.* [15] in 2008, Liu and Fang [8] in 2007, and Temudo *et al.* [35] in 2008. This observation was supported by the drop of pH from 6.8 to 6.6 observed between 12 and 24 h (Figure 6A) and yielding significant amount of CO_2_ and decreased oxygen release (Figure 5B). Glycerol conversion also supported cell growth, resulting in an increase in cell density (CD) measured as an increase in optical density of about 0.40 at 600 nm (Figure 5C). This is in agreement with Murarka *et al.* [36], who reported that 20% of the carbon incorporated into the protein fraction of biomass originated from glycerol. For the remaining period of time in batch mode, pO_2_ dropped and approached zero again (Figure 5A) while *E. aerogenes* was still expanding its population (from 0.60 to 0.83) and pO_2_ started to increase slightly again when cell density stabilized. After about 24 to 48 h, when nitrate and nitrite were consumed (<36% nitrite and non detectable nitrate) cell density started to drop from 0.83 to 0.73 (Figure 5C). Under these conditions of nitrogen and O_2_ limitation, *E. aerogenes* switched its metabolism mode from semi-aerobic to anaerobic conditions. This resulted in no further expansion of the population (dropped from 0.83 to 0.73), increased hydrogen yield (0.30 to 0.62 mole/mole GL), increased glycerol conversion (0.34 to 0.47) and increase ethanol production (0.03 to 0.31 mole/mole GL).

At 72 h, the reactor operation was switched to continuous mode by feeding fresh glycerol-containing media. This resulted in a significant increase in glycerol conversion (0.47 to 0.97), ethanol yield (0.31 to 0.67 mole/mole glycerol) and hydrogen yield (0.62 to 0.67 mole/mole GL). However, cell density continued to drop, from 0.73 to 0.65 (Figure 5C), indicating a significant dilution effect due to the fresh feed. Liquid recycling was then started at 96 h. Constant product concentrations were obtained at 144 h onwards and the operation of the bioreactor was kept stable for two days (until 192 h). Over this period of steady operation, nitrate was totally consumed; the nitrite residual concentration was down to 25%; pO_2_ was reduced and stabilized at 19%; and cell density was stable to 0.83. Glycerol conversion was almost complete and resulted in yields of hydrogen and ethanol of 0.86 and 0.75 mole/mole GL, respectively.

## 3. Experimental Section

### 3.1. Microorganism and Inoculum Preparation

*Enterobacter aerogenes* (ATCC 35029) was obtained from American Type Culture Collection (ATCC). The culture was started under aerobic condition in 100 mL of nutrient broth BD 234000 from Becton and Dickinson Company (12 g/L), incubated at 37 °C and 120 rpm for 20 to 24 h to reach stationary phase. Two different types of inoculum bottles (250-mL and 500-mL inoculum bottles) were prepared from this initial aerobic culture by transfer of 5% vol inoculums. Inoculums for the experiments conducted in bottles were prepared by transferring 10-mL of seeding into 190 mL of nutrient broth contained in 250-mL serum bottles. Inoculums for experiments conducted in the bioreactor were prepared by transferring 14 mL of seeding into 282 mL of nutrient broth contained in 500-mL serum bottles. These bottles were incubated under semi-anaerobic conditions at 37 °C and 120 rpm for 20 to 24 h to reach stationary phase.

### 3.2. Minimum Mineral Synthetic Media (MMSM) and Glycerol Concentration

MMSM formulation was obtained from Jitrwung and Yargeau 2010 [19] and prepared using the following amounts per litre of deionised water: ammonium nitrate (NH_4_NO_3_: 1.5 g), disodium hydrogen phosphate (Na_2_HPO_4_: 12.2 g), magnesium sulfate heptahydrate (MgSO_4_∙7H_2_O: 200 mg), ferrous sulfate heptahydrate (FeSO_4_∙7H_2_O: 6.25 mg) obtained from Sigma Aldrich and tetraethylenediamine disodium salts (Na_2_EDTA: 3.5 mg) obtained from Fisher Scientific. Crude glycerol (CG) obtained from Rothsay Biodiesel Canada was first vacuum filtered using filter paper P8 and P4 purchased from Fisher Scientific and then dissolved in deionized water in order to obtain a concentration 18.5 g crude glycerol/L, equivalent to 15 g pure glycerol/L. In all experiments, pH of the solution was adjusted to an initial value of 6.8 by using 10% of phosphoric acid obtained from Fisher Scientific. For the experiments conducted in bottles, the amount of oxygen in the glycerol-MMSM mixture was reduced by boiling the solution for 20 min, cooling down for 5 min, and then cooling down on ice for 5 min with a continuous argon flushing of the headspace [19]. For the experiments conducted in the bioreactor, argon was used to purge the system until the dissolved oxygen concentration (pO_2_) was measured as 0%.

### 3.3. Biohydrogen Production Experiments

#### 3.3.1. Biohydrogen Production in the Experiment Bottles

At the stationary phase, the 250-mL inoculum bottles were flushed with an argon/oxygen gas mixture (7.5% O_2_) to obtain semi anaerobic conditions. Using the Hungate technique [37], these bottles were then slightly over-pressurized with the same gas mixture prior to removing the 9.4-mL inoculum to be transferred to 50 mL of the MMSM/glycerol solution placed in a 125-mL serum bottle; referred to as the experiment bottles. The experiment bottles were then placed in the incubator shaker at 37 °C and 120 rpm until hydrogen production ceased.

#### 3.3.2. Biohydrogen Production in the Bioreactor

The 500-mL inoculum bottles were flushed with an argon/oxygen gas mixture (7.5% O_2_) to obtain semi anaerobic conditions. The entire content of the inoculum bottle (282 mL of solution) was then fed to the bioreactor containing 1500 mL of MMSM/glycerol solution, using a peristaltic pump while replacing the liquid with an equivalent volume of the same gas mixture (7.5% O_2_). A 3.6-L Labfors 4 bioreactor from Infors HT (working volume of 1.8 L) was used in batch and continuous mode of operation at a temperature 37 °C. In batch mode, mixing speed was set to 500 rpm and hydrogen production was monitored off-line until production ceased. Figure 6 provides an overview of the setup designed for the continuous mode. The configuration allowed, in addition to the previously mentioned parameters, monitoring of dissolved oxygen (using a pO_2_ sensor probe, oxygen FDA 325, sensor NTC: 22 kOhm obtained from Hamilton) and gas production as well as control, using peristaltic pumps, over pH using 10% phosphoric acid or 10% sodium hydroxide, feed rate, liquid recycling and waste recycling (to evaluate potential savings by recycling nutrients).

### 3.4. Analytical Methods

#### 3.4.1. Gas Analysis

Batch mode: The volume of gas produced was measured using a gas-tight syringe every 6 h for experiments performed in the serum bottles and the bioreactor operated in batch mode. The concentration of hydrogen was monitored by gas chromatography (GC) using a Hewlett Packard 5890 equipped with a molecular sieve column 8A maintained at a temperature of 80 °C and a thermal conductivity detector (TCD) and using 3.0 mL/min argon as a carrier gas.

Continuous mode: The volume of gas produced was monitored using a gas flow meter (E1 in Figure 6). The concentration of hydrogen was monitored by online gas chromatography (GC) using a SRI 8610C Multiple Gas Analyzer equipped with two columns: a 6' Silica Gel and a 6' Molecular Sieve column. The initial temperature of 40 °C was hold for 4 min and ramped at 20 °C/min, 20 mL/min of helium was used as a carrier gas and two detectors were used: a thermal conductivity detector (TCD) and flame ionized detector (FID). The gas analysis was performed every 2 h with an analysis time of 12 min and post run time of 108 min.

#### 3.4.2. Liquid Analysis

10-mL aliquots of the residual liquid were collected when the hydrogen production ceased. The liquid samples were centrifuged at 10,000 rpm for 20 min. The supernatant was filtered through 0.2 μm syringe filters and then analysed by liquid chromatography and ion chromatography. A Hewlett Packard 1050 liquid chromatograph, equipped with a Rezex ROA-Organic Acid H^+^ 8% 150 × 7.80 mm column and a refractive index detector (RI) HP1047A, was used to measure the concentration of glycerol, 1,3 propanediol, pyruvate, lactate, acetate, formate, 1-propanol, methanol, and ethanol. The mobile phase was 0.035 M H_2_SO_4_ at a flow rate of 0.8 mL/min. The column temperature was 65 °C and the temperature of RI was 50 °C. Inorganic ions, including nitrite, nitrate, sulfate, and phosphate were monitored using an ion chromatograph (IC) equipped with a Metrosep A supp7 250/4.0 mm-5 μm column maintained at 45 °C and a conductivity detector. 3 mM Na_2_CO_3_ was used as the mobile phase.

#### 3.4.3. Monitoring of Cell Growth

In order to monitor cell growth, 3-mL aliquots were collected every 6 h from the CSTR, every 24 h from the bioreactor operated in batch mode and when hydrogen production ceased for the serum bottles. An Evolution 300 UV-visible Spectrometer was used to measure the optical density at 600 nm.

## 4. Conclusions

Crude glycerol can be converted to biohydrogen and bioethanol by *E. aerogenes* using optimized media composed of Na_2_HPO_4_, NH_4_NO_3_, MgSO_4_, FeSO_4_, and Na_2_EDTA and optimized operating conditions: pH at 6.4, temperature 37 °C, mixing speed 500 rpm, fresh feed rate 0.44 mL/min, liquid recycling ratio 0.33 and glycerol concentration 15 g glycerol/L. Under these conditions, stable production was obtained, resulting in yields of hydrogen at 0.86 mole/mole GL and ethanol at 0.75 mole/mole GL. These yields obtained for crude glycerol are higher than most values reported in literature using pure and crude glycerol and various strains of *E. aerogenes* (summarized in Table 1), except for the results reported by Ito *et al.* in 2005. However, a great benefit of the work reported here is the much lower media cost (0.91$/L compared to close to 4$/L), in addition to the advantages of the continuous and stable production offered by the CSTR used here over the batch system used in previous study by Ito *et al.* [20] which reported higher yields. Lastly, this process shows great economic potential due to the production of two value-added products, hydrogen and ethanol, and the possibility of using the ethanol produced as a raw material in biodiesel production.

**Table 1:** Comparison of media cost, hydrogen and ethanol yields for the conversion of crude glycerol in batch and continuous systems using various strains of *Enterobacter aerogenes*.

Yields (mole/mole GL)		Initial Glycerol Concentration (g/L)	Reactor Type	$CAD/L of Media	Reference
H_2_	Ethanol				
1.12	0.96	1.7	Batch	3.68	Ito *et al.* 2005 [20]
0.96	0.90	15	Batch–3.6L	0.91	This study
0.86	0.75	15	CSTR	0.91	This study
0.74	0.92	10	Bioelectrochemical	Not reported	Sakai and Yagishita 2007 [21]
0.12	0.83	31	Batch	Not reported	Reungsang *et al.* 2013 [38]

## Supplementary Materials

Supplementary materials can be found at http://www.mdpi.com/1422-0067/16/05/10650/s1.
