# Supplementary material for: Biohydrogen and Bioethanol Production from Biodiesel-Based Glycerol by Enterobacter aerogenes in a Continuous Stir Tank Reactor
Source: Int J Mol Sci. 2015 May 11;16(5):10650–64. doi: 10.3390/ijms160510650 (PMC4463667; doi:10.3390/ijms160510650)
Supplement: Supplementary file 1 [file ijms-16-10650-s001.pdf]

Supplementary Information

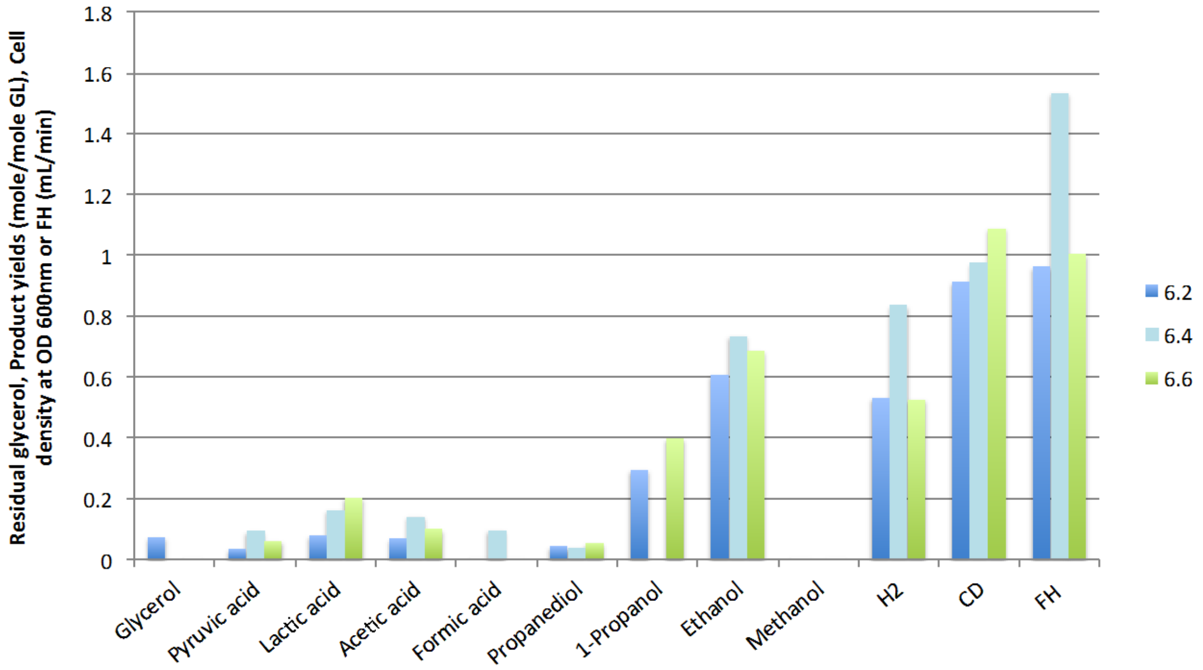

Figure S1. Effect of pH on residual glycerol, hydrogen, ethanol and other product yields, cell density, and rate of hydrogen production (FH) (3 replicates).

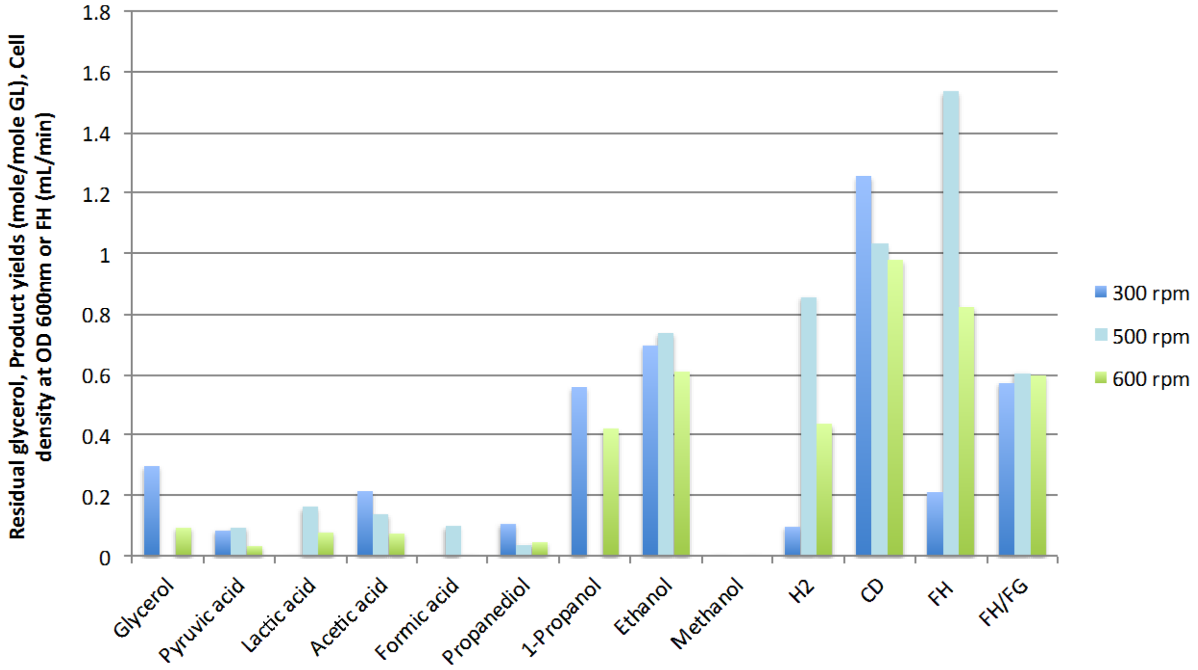

Figure S2. Effect of mixing speed on residual glycerol, hydrogen, ethanol and other product yields, cell density and rate of hydrogen production (FH) (3 replicates).

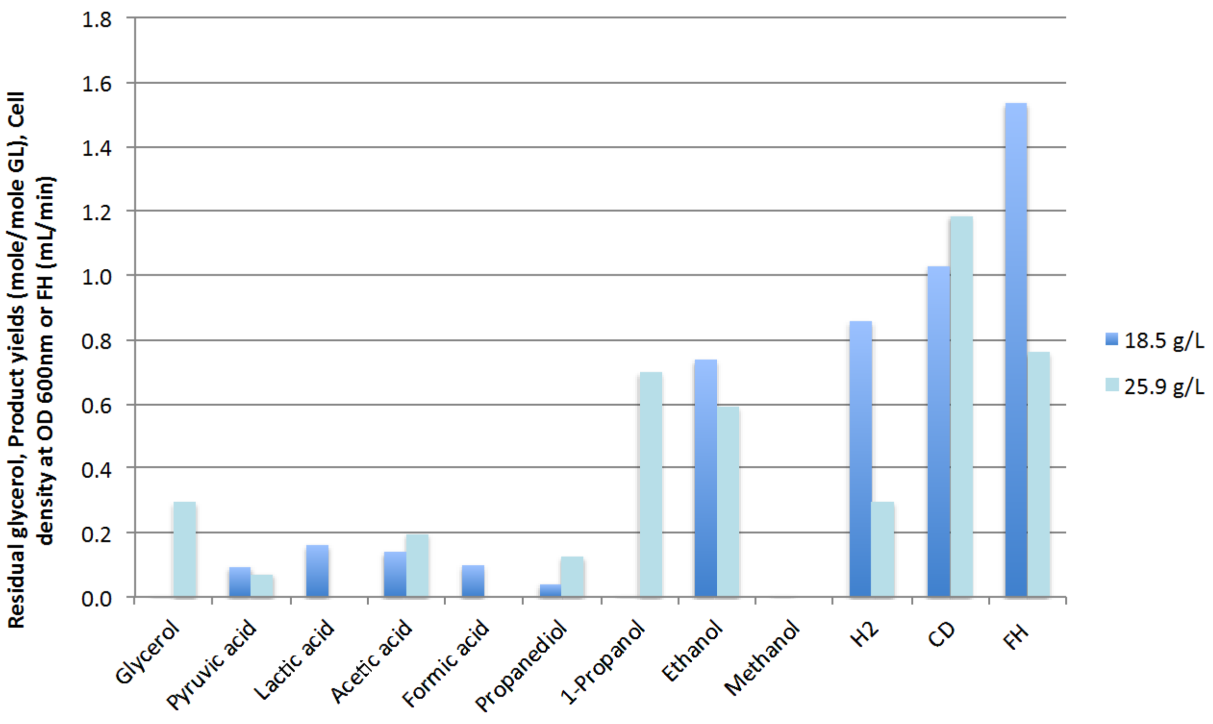

Figure S3. Effect of glycerol concentration on residual glycerol, hydrogen, ethanol and other product yields, cell density and rate of hydrogen production (FH) (3 replicates).

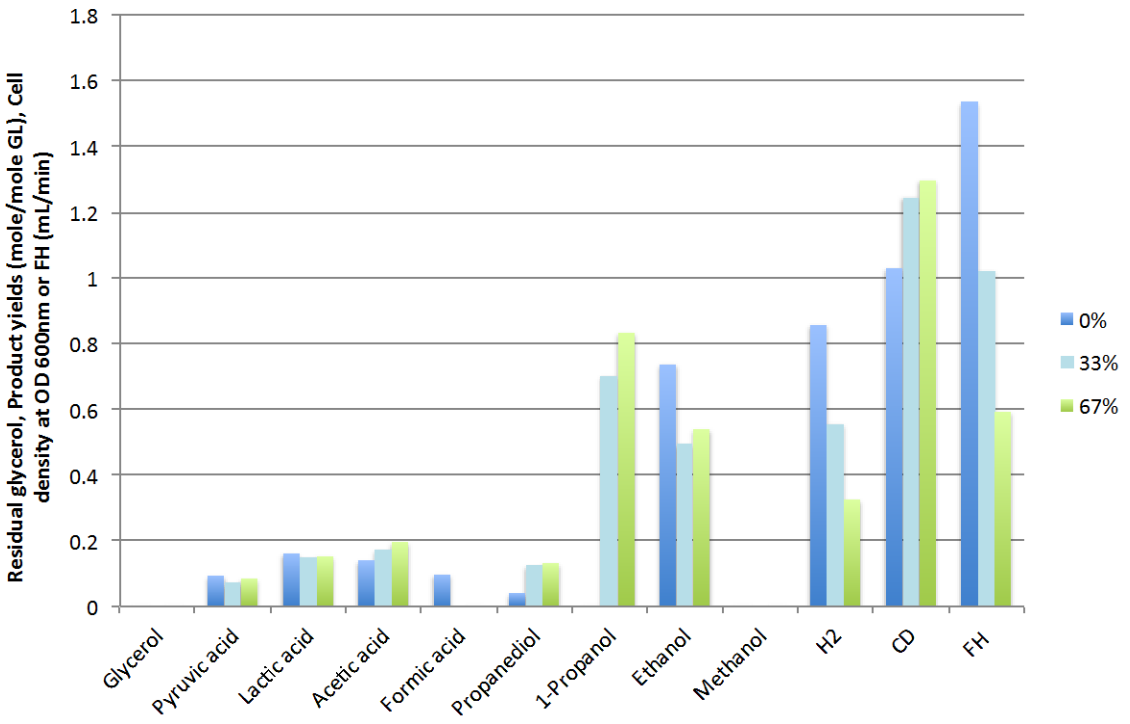

Figure S4. Effect of waste recycling on residual glycerol, hydrogen, ethanol and other product yields, cell density and rate of hydrogen production (FH) (3 replicates).
